# Framing Patellar Instability: From Diagnosis to the Treatment of the First Episode

**DOI:** 10.3390/jpm13081225

**Published:** 2023-08-02

**Authors:** Davide Maria Maggioni, Riccardo Giorgino, Carmelo Messina, Domenico Albano, Giuseppe Michele Peretti, Laura Mangiavini

**Affiliations:** 1 Residency Program in Orthopaedics and Traumatology, University of Milan, 20122 Milan, Italy; davidemaria.maggioni@unimi.it; 2 IRCCS Istituto Ortopedico Galeazzi, 20157 Milan, Italy; carmelo.messina@unimi.it (C.M.); albanodomenico.md@gmail.com (D.A.); giuseppe.peretti@unimi.it (G.M.P.); laura.mangiavini@unimi.it (L.M.); 3 Dipartimento di Scienze Biomediche per la Salute, Università degli Studi di Milano, 20122 Milan, Italy; 4 Dipartimento di Scienze Biomediche, Chirurgiche ed Odontoiatriche, Università degli Studi di Milano, Via della Commenda 10, 20122 Milan, Italy

**Keywords:** patella, instability, diagnosis, treatment, knee

## Abstract

The patellofemoral joint (PFJ) is a complex articulation between the patella and the femur which is involved in the extensor mechanism of the knee. Patellofemoral disorders can be classified into objective patellar instability, potential patellar instability, and patellofemoral pain syndrome. Anatomical factors such as trochlear dysplasia, patella alta, and the tibial tuberosity–trochlear groove (TT-TG) distance contribute to instability. Patellofemoral instability can result in various types of dislocations, and the frequency of dislocation can be categorized as recurrent, habitual, or permanent. Primary patellar dislocation requires diagnostic framing, including physical examination and imaging. Magnetic resonance imaging (MRI) is essential for assessing the extent of damage, such as bone bruises, osteochondral fractures, and medial patellofemoral ligament (MPFL) rupture. Treatment options for primary dislocation include urgent surgery for osteochondral fragments or conservative treatment for cases without lesions. Follow-up after treatment involves imaging screening and assessing principal and secondary factors of instability. Detecting and addressing these factors is crucial for preventing recurrent dislocations and optimizing patient outcomes.

## 1. Introduction

The patellofemoral joint (PFJ) is a complex articulation between the patella and the femur [1,2]. The patella is the largest sesamoid of the human body. It is involved in the extensor mechanism bridging the quadriceps femoris to the proximal tibia, thus generating a transferring of forces necessary to knee extension [3].

On the other hand, the femur, with its trochlear groove, accommodates the patella. At full knee extension, the patella lays above the trochlear groove. In contrast, as the knee reaches around 30° of flexion, the patella starts engaging the trochlear groove, progressively increasing lateral translation and lateral patellar tilt [3,4,5]. We are therefore faced with a foul of force distribution. Anatomical structures, such as bone morphology and ligaments, in combination with adequate neuro-muscular control, play a decisive role in controlling patella–femoral kinematics [6]. Patellofemoral disorders may result.

## 2. Classification of Patellofemoral Disorders

The Lyonnaise school has perfected a simple and easily applicable classifying system for patellofemoral pathology by dividing it into three main groups, as follows [7]:

- *Objective patellar instability*: Patients have experienced at least one episode of patellar dislocation and present at least one or more principal factors of instability. Patellar dislocation can occur during high-energy activities such as sports and is frequently associated with hemarthrosis.
- *Potential patellar instability*: Patients never experienced a true patellar dislocation, although they report a rather generic feeling of instability. It may occur daily during low-demand activities, such as walking and climbing stairs. These patients present one or more principal factors of instability.
- *Patellofemoral pain syndrome*: Pain is the main symptom, and it is mostly attributed to cartilage wear on either the patellar or femoral side. Imaging does not show any evident factor of instability, nor can a clinical episode of patellar dislocation be identified. In subjects suffering from patellofemoral pain (PFP) syndrome, exercise therapy should be considered the first-line treatment option as it is considered the “treatment of choice” and is supported by high-level evidence. Such therapy should include exercises for strengthening the hip and knee; these exercises can be performed through kinetic chain exercises (either weight-bearing or non-weight-bearing). Additionally, joint mobilization targeted at the knee, patellofemoral taping, and neuromuscular training have also been suggested as second-line treatment options to be used in conjunction with exercise therapy [8].

## 3. Principal Factors of Instability

**Figure 1:**
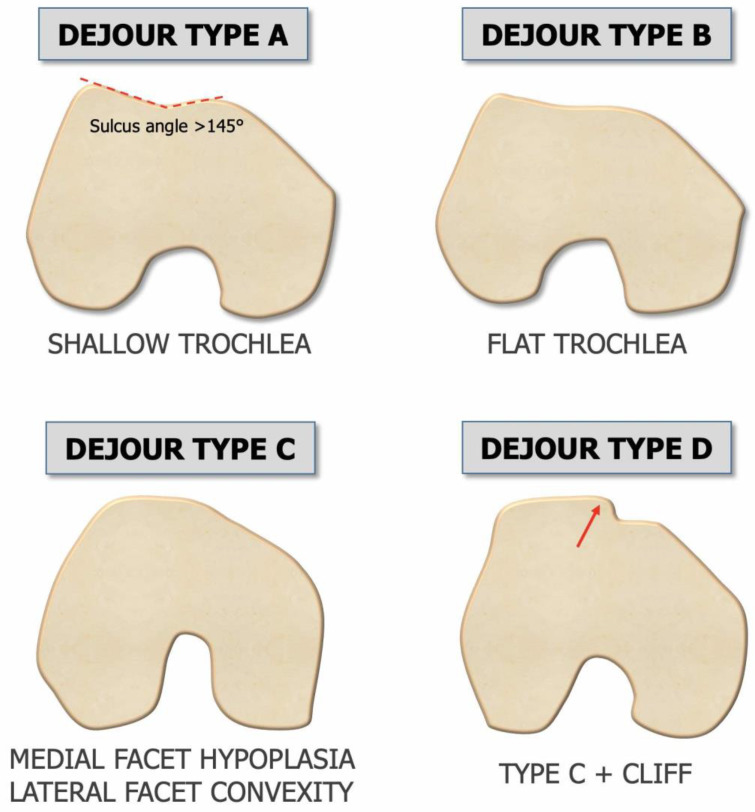
Dejour classification of trochlear dysplasia on axial view [7]. (Type A) Flattening of the trochlea (sulcus angle > 145°) is observed, but concavity is preserved. (Type B) The lateral facet is flat to convex with a possible supratrochlear spur. (Type C) The medial facet is hypoplastic, and the lateral facet is convex. (Type D) Complete flattening of the trochlea, with a marked depression on the medial facet (cliff sign).

**Figure 2:**
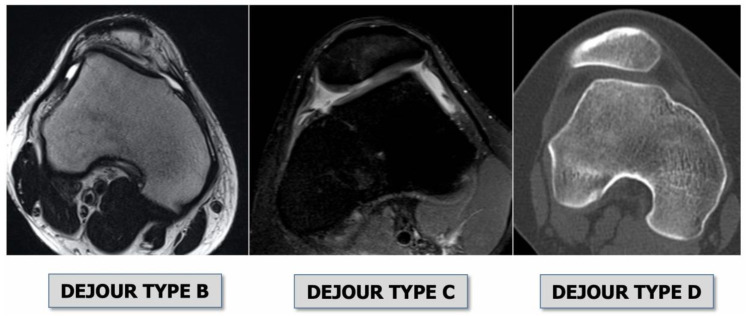
Types trochlear dysplasia on MRI axial view, as classified by Dejour [7].

**Figure 3:**
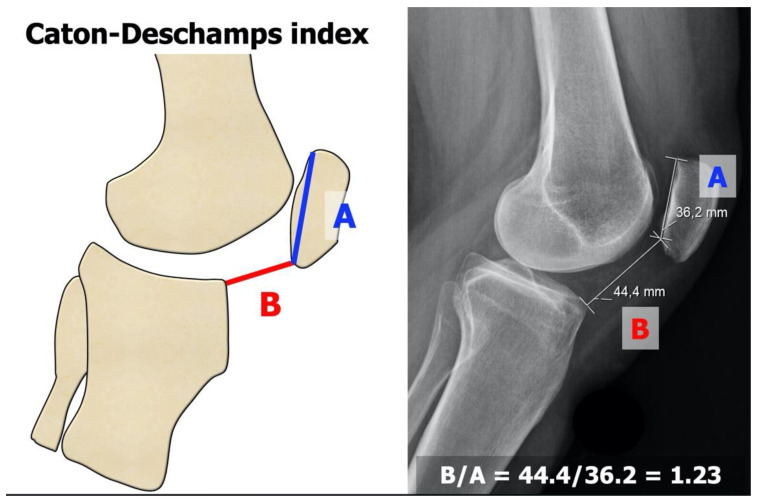
Caton–Deschamps Index (CDI) [10,11]. The CDI corresponds to a ratio (B/A) of the distance between the tibial plateau anterior angle to the patellar articular surface lowest aspect (B) and the patellar articular surface length (A).

**Figure 4:**
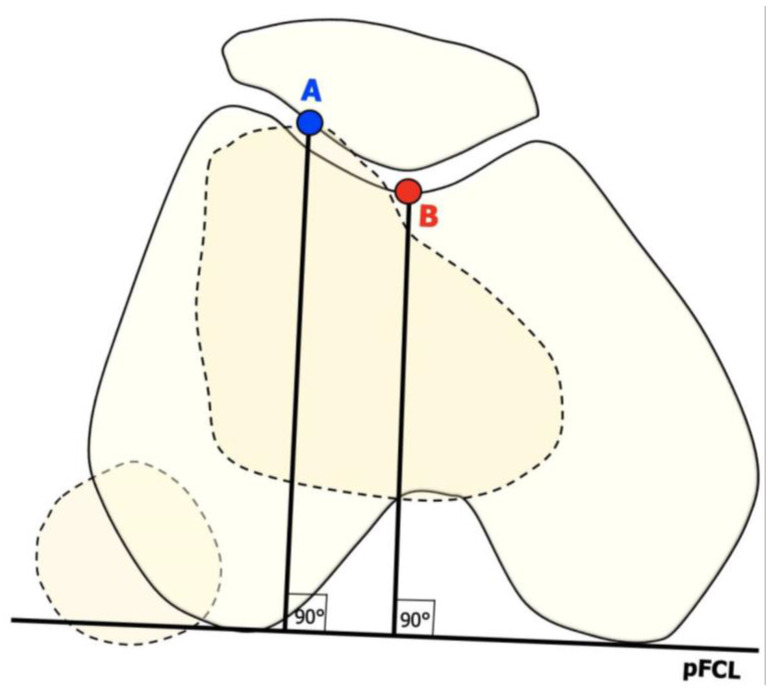
TT-TG distance. (A) The most prominent point of tibial tuberosity (TT). (B) Trochlear groove (TG).

**Figure 5:**
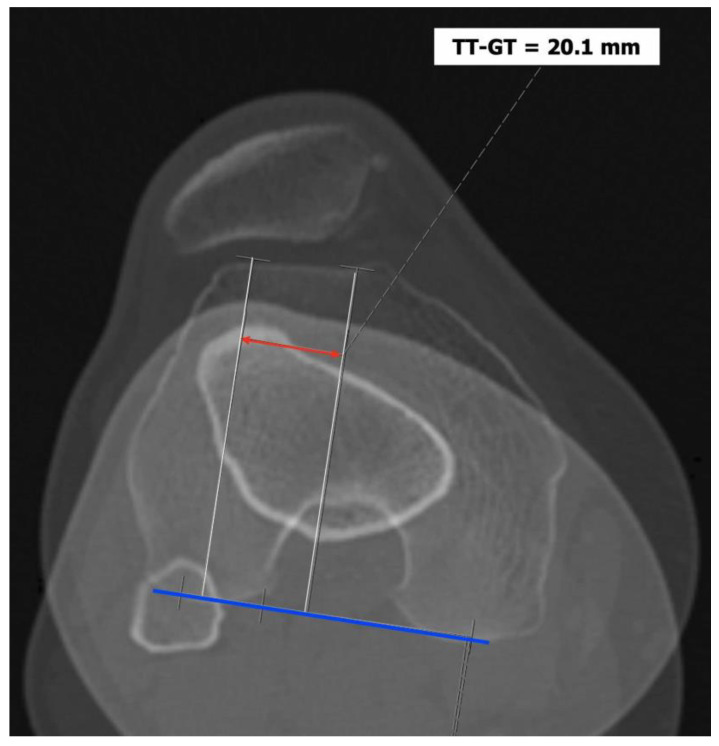
CT scan with pathological TT-TG of 20.1 mm.

Three main features were revealed to be relevant in knees with patellar instability that basically depend on anatomical parameters and thus on bone morphology [7]:

1. *Trochlear dysplasia* indicates whether the femoral trochlea is flat or convex (instead of concave), causing abnormal patellar tracking and a loss of joint congruence (Figure 1 and Figure 2). Dejour classified trochlear dysplasia into four groups [9]. This classification system requires an accurate lateral X-ray (congruent posterior condyles) and confirmation via axial imaging of the knee (CT scan or MRI). A certain degree of trochlear dysplasia was found in up to 96% of patients with objective or potential patellar instability [7].
2. *Patella alta* is defined as an excessive patellar height that prevents or limits patellar engagement on the trochlea during flexion, thus predisposing the patient to patellofemoral instability. It is easily measured using the Caton–Deschamps Index (CDI) on an accurate lateral knee X-ray [10,11]. It is pathological when the CDI is greater than or equal to 1.2 (Figure 3). It is present in 30% of patellar dislocations [12].
3. *The tibial tuberosity-trochlear groove (TT-TG) distance* [13,14] is defined as the transverse length between the most prominent point of the tibial tuberosity and the trochlear groove on the femur, calculated on axial images (CT scan or MRI), representing the axial malalignment of the extensor mechanism. The greater the distance, the greater the lateralizing force acting on the patella. It is pathological when TT-TG > 13 mm on MRI or TT-TG > 20 mm on a CT scan (Figure 4 and Figure 5).

## 4. Patellofemoral Instability

When patients present an anatomical predisposition (at least one principal factor of instability), the patella may incur dislocation. In general, the patella dislocates laterally as the extensor mechanism’s biomechanics push toward the knee’s lateral side. Other rare cases of dislocations are classified depending on the patella’s position, such as medial dislocation, which is usually iatrogenic, vertical intercondylar dislocation, or horizontal intra-articular dislocation [15,16].

Patellofemoral instability can be further classified into the following categories according to the frequency of dislocation:

- Recurrent, when the patella dislocates frequently during knee flexion (two or more episodes are necessary) [17];
- Habitual, when the patella dislocates every time the knee flexes in early knee flexion (<30°) and spontaneously relocates with the extension of the knee [18].
- Permanent, when the patella is permanently dislocated through the entire knee range of motion, never facing the trochlea [19].

Dislocation is caused by a concomitance of factors, such as sports trauma, genetics, and age, to which anatomical predisposition is added.

Patellofemoral dislocation accounts for 3% of knee traumas. The average annual incidence of primary patellar dislocation has been reported to be 5.8 cases per 100,000 in the general population, with the highest incidence occurring in the second decade of life (29 per 100,000) [20]. It is more common in females and may be associated with other injuries within the knee [21]. The rate of recurrence can be up to 15–44%, and patients with a history of two or more dislocations have a 50% chance of recurrent dislocation episodes, meaning that an important slice of the population that undergoes primary patellar dislocation will not experience recurrence [22].

In 2016, Schmeling and Frosch introduced a new classification for patellar instability and maltracking, with the aim of taking into consideration both clinical and radiological pathologies [23]. This classification is based on “instability” criteria, but it also introduces the evaluation of “maltracking” criteria and “loss of patellar tracking”; overall, these factors are evaluated via both clinical and radiological aspects. Maltracking is further divided into two subtypes. According to the combination of the above factors and based on the main pathology, five types of patellar instability and maltracking are identified:

- Type 1: patellar dislocation after trauma, without instability and without patella maltracking.
- Type 2: patella instability without clinical or radiological signs of patella maltracking.
- Type 3: a combination of patella instability and patella maltracking. This type is divided into four subtypes, according to the main cause of the maltracking: (a) soft tissue contracture; (b) patella alta; (c) an abnormal tibial tuberosity–trochlea groove distance; (d) valgus deviations; and (e) torsional deformities.
- Type 4: instability and maltracking with a loss of patella tracking due to severe trochlear dysplasia, leading to a highly unstable ‘‘floating patella”.
- Type 5: maltracking without instability.

This classification is advantageous because not only allows for a clear discussion of the specific case but can also be helpful in making therapy decisions as it provides surgical options for each type (and subtype).

## 5. Primary Dislocation: Diagnostic Framing

A patient suffering from primary patellar dislocation refers to the emergency room with a painful knee and possible flexion inability. After plain knee radiographs and an axial view of the patella, the physician performs a reduction maneuver. The patient may also arrive at the emergency room with the patella already relocated, only complaining of a painful, swollen knee. Upon physical examination, typical findings are medial side tenderness due to medial patello-femoral ligament (MPFL) rupture and a swollen knee, which may require aspiration. Patellar dislocation is a common cause of knee hemarthrosis, especially in adolescents [24].

On the X-rays, trauma surgeons must focus their attention on the following:

- Bony avulsions: depending on the size, they may require surgery [25];
- Patella alta: the CDI must be calculated. It is often the first sign of a possible PF disorder;
- Trochlear dysplasia: the crossing sign, the supra-trochlear spur, and the double contour should be identified for classification according to Dejour [9].

**Figure 6:**
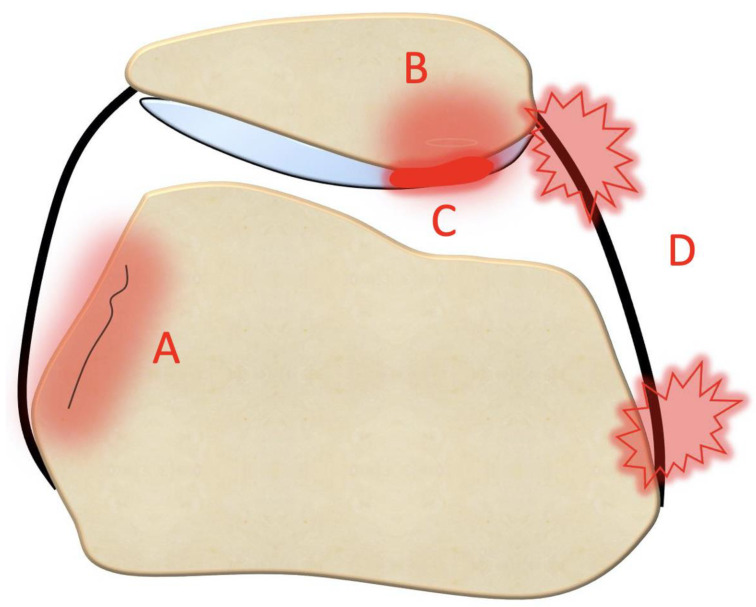
Consequences of acute patellar instability on the knee: (A) lateral condyle bone bruise. (B) medial patellar bone bruise. (C) patellar osteochondral damage. (D) MPFL rupture and its location, patellar insertion, mid-substance, or femoral insertion. Performing MRI within a few days from the injury is crucial to assess the patellar–femoral damages.

**Figure 7:**
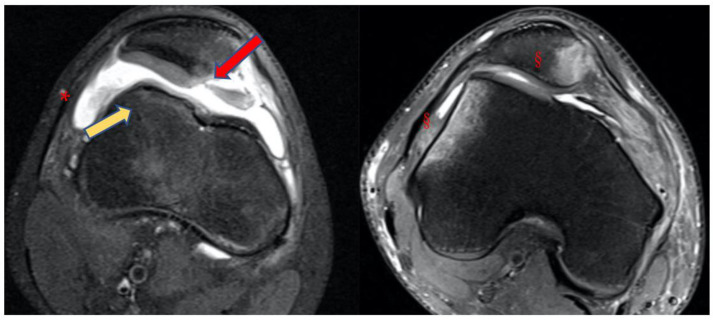
Case of a primary patellar dislocation, shown in MRI axial views. Note the large amount of synovial fluid or hemarthrosis (*), the bone bruise on its typical presentation (§), the chondral defect on the medial side of the patella (red arrow), and the shape of the trochlea (yellow arrow).

An MRI within a few days after the trauma is highly recommended in addition to plain radiographs [26]. Indeed, MRI allows for the detection of the following (Figure 6 and Figure 7):

- Bone bruises on the medial side of the patella and the lateral condyle, which indicate with certainty the occurrence of a recent patellar dislocation;
- Osteochondral fracture with possible loose bodies, which are important to rule out, especially in skeletally immature patients. If the osteochondral fracture has a sufficient size (5–10 mm on MRI), urgent reduction and fixation, either open or arthroscopically, must be considered [25];
- Trochlear dysplasia, for which axial images are needed to achieve a correct classification according to the Dejour classification [9];
- MPFL rupture and location. Typically, after a patellar dislocation, the MPFL is torn [26,27]; therefore, the patella loses its major soft tissue stabilizer, which may lead to recurrent patellar instability [28].

MRI remains necessary in cases of suspected primary patellar dislocation to make a correct diagnosis, to assess concomitant osteocartilaginous injuries, and to evaluate principal factors of instability [29,30]. Recognizing the abnormalities in the bony anatomy following a primary dislocation is essential to treating patellar dislocation.

After the first episode, a meticulous physical examination and sufficient imaging modalities are crucial in identifying patients at a high risk of recurrence [31,32]. Skeletally immature patients are also at a higher risk of recurrence than adults. Balcarek et al. [33] proposed the “Patellar Instability Severity Score” (PISS) as a practical tool for initial risk assessment, helping to discriminate between the patients at a low risk of recurrence (PISS ≤ 3) and those at a high risk of recurrence (PISS ≥ 4, associated with a risk of recurrent dislocation up to five times greater) (Table 1). PISS is not a therapeutic algorithm; nevertheless, it may aid the orthopedic surgeon in correctly informing the patient of their condition and in being more inclined to choose a conservative or surgical approach [34]. In our opinion, it remains essential to choose an approach tailored to the patient and their condition, avoiding the so-called one-for-all approach.

## 6. Primary Dislocation: Treatment Options

### 6.1. Urgent Surgery

Primary dislocation with a patellar osteochondral fragment is the only indication for urgent surgery [35]. The surgery consists of osteochondral fixation with pins, screws, or darts, whether absorbable or not absorbable. The dimensions of the osteochondral lesion should allow for the use of the fixation devices. MPFL reconstruction is not indicated. After surgery, an immediate rehabilitation protocol is essential. Several rehabilitation protocols exist, and they can differ according to the center and surgeon. A possible proposed protocol includes full weight bearing as the osteochondral lesion is not located in a loading zone, thereby minimizing any impact on the osteochondral fixation. Crutches are recommended for 30 days, while no brace is required. Isometric exercises for the quadriceps should be performed. Additionally, a progressive passive motion from 0° to 100° is advised for 45 days. It’s important to note that the flexion of the knee can engage the patella on the trochlea, potentially affecting the osteochondral fixation. Therefore, passive motion should be stepwise to prevent any adverse effects on the fixation, though it is still necessary to avoid articular stiffness.

### 6.2. Conservative Treatment

In cases of dislocation without osteochondral lesions, surgery is not indicated. Arthrocentesis may relieve the patient’s symptoms. Arthrocentesis also reduces the tension on the capsule and MPFL, allowing for better patellar position and decreasing pain. After surgery, an immediate rehabilitation protocol is implemented to facilitate the recovery process. This protocol includes several components:

Firstly, ice therapy is applied to help reduce swelling and manage pain in the affected area. It is typically administered intermittently for short durations. Full weight bearing is encouraged unless the osteochondral lesion is situated in a loading zone. In such cases, partial weight bearing or non-weight bearing for the first weeks may be advised to protect the affected area. Crutches are utilized for a duration of 30 days to assist with mobility and alleviate pressure on the healing joint. Light patellar bracing is employed to limit tension on the medial patellofemoral ligament (MPFL), supporting its healing and stability during the initial stages of rehabilitation. Isometric exercises of the quadriceps are prescribed to maintain muscle strength and promote stability around the knee joint. Finally, progressive passive motion exercises ranging from 0° to 100° of flexion are performed over a period of 45 days. This gradual increase in motion helps prevent joint stiffness and improve the range of motion, ensuring optimal recovery. Overall, this comprehensive rehabilitation protocol aims to promote healing, restore function, and minimize complications following surgery for patellar instability.

## 7. Primary Dislocation: Follow-Up

Follow-up may be scheduled 45 days after injury, with complete imaging screening: X-rays, MRI, or a CT scan. Regarding second-level imaging, MRI is generally recommended when ligamentous injury is suspected. On the other hand, when bony damage is suspected (with possible presence of osteochondral loose bodies), a CT scan is more appropriate [36]. On this occasion, the orthopedic surgeon will assess the effects of the conservative treatment and explain the risk factors for recurrence to the patient by identifying and quantifying the principal factors of instability and the PISS score. A return to sport is allowed three months after injury. In addition to the primary factors of instability, it is important for trauma surgeons to also focus on the so-called “secondary factors” of instability. These secondary factors encompass various aspects that can contribute to instability in the knee joint. One of these secondary factors is varus/valgus malalignment of the knee, which refers to the deviation of the knee joint from its normal alignment. This misalignment can place additional stress on the joint and contribute to instability. Another secondary factor is genu recurvatum, which is characterized by the hyperextension of the knee joint beyond its normal range. This excessive extension can compromise the stability of the knee and increase the risk of instability. Torsional malalignment of the lower extremities is also an important secondary factor to consider. This involves the abnormal rotation of the femur or tibia, such as increased femoral anteversion or increased internal torsion of the distal femur. These rotational abnormalities can affect the alignment and stability of the knee joint. Patellar dysplasia, classified by the Wiberg system [37], is another secondary factor that can contribute to instability. Patellar dysplasia refers to abnormal development or shape of the patella, which can affect its tracking and stability within the patellofemoral joint. Lastly, abnormal pronation of the subtalar joint can also be a secondary factor of instability. Excessive inward rolling of the foot during walking or running can impact the alignment and stability of the entire lower extremity, including the knee joint.

While the principal factors of instability must be detected via an accurate instrumental evaluation (MRI or CT scan), the secondary factors of instability can initially be assessed clinically. The physical examination starts by analyzing walking and standing positions, focusing on varus/valgus malalignment, torsional malalignment, and increased foot pronation. Any clinically evident torsional malalignment of the lower extremities, such as increased femoral anteversion or increased internal torsion of the distal femur, can be better defined via specific imaging evaluation (CT scan). Patellar dysplasia, classified via the Wiberg system, can be assessed on an axial view of radiographs of the patella. Abnormal pronation of the subtalar joint may require correction with orthotics. It is also suggested to examine the patient seated with their lower legs hanging. In many cases, when asked to actively extend the leg, a patient with patellar instability may present a so-called J sign, which means the patella moves resembling an inverted J while extending the knee. The apprehension test is positive in the majority of patients with patellar instability: the physician gently pushes the patella toward lateral at 0°–30°–60° and 90° of flexion with relaxed quadriceps. Avoidance or protective quadricep contraction indicates a positive test [38,39]

Patellar instability at 0°–30° of knee flexion reflects an insufficiency of passive stabilizers (mostly MPFL); instability at 0°–60° of knee flexion depends on an insufficiency of passive and often of static stabilizers (pathological TT-TG, trochlear dysplasia, patella alta, and valgus knee). Patellar instability at 0°–90° of knee flexion relies on complex rotational bony malalignment.

The study of the primary and secondary factors of instability gives us a complete framework of primary patellar instability. In the absence of osteochondral fractures, the treatment of primary patellar instability is usually conservative. If recurrence is highly probable (PISS ≥ 4) or important rotational malalignment is present, operative treatment should be considered. Considering the severity of the deformity, several surgical treatments can be taken into consideration. These include both arthroscopic and open MPFL repair or reconstruction, which have shown promising outcomes in recent studies [40,41,42]. Additionally, Fulkerson-type osteotomy has emerged as an effective approach in addressing complex cases [43]. Moreover, trochleoplasty has shown positive results in managing patellofemoral instability and can be considered a viable option for specific cases [43]. Lastly, the Goldthwait technique has also been explored as a potential treatment for patellofemoral instability and warrants consideration based on individual patient factors [44,45].

Understanding why the patient underwent primary patellar dislocation is a task for the orthopedic surgeon, allowing them to adopt the best therapeutical approach.

The prognosis of patellar instability can vary according to the disease course. After a first-time dislocation, nearly half of the subjects may experience further dislocations. A history of chronic instability with recurrent dislocations may lead to progressive cartilage damage, thus potentially predisposing individuals to post-traumatic arthritis.

## 8. Conclusions

Patellofemoral instability can lead to different types of dislocations, making it necessary to establish an accurate diagnosis for primary patellar dislocation. The use of MRI is crucial to assess the extent of damage and determine the appropriate treatment options. Additionally, thorough post-treatment follow-up is essential, involving the analysis of both primary and secondary factors of instability. The orthopedic surgeon’s role is to understand the underlying cause of the patient’s primary patellar dislocation.

## Figures and Tables

**Table 1:** Patellar Instability Severity Score.

Age	
>16	0
≤16	1
Bilateral instability	
No	0
Yes	1
Trochlear dysplasia	
None	0
Mild (type A)	1
Severe (type B–D)	2
Patellar height, IS ratio	
≤1.2	0
>1.2	1
TT-TG distance	
<16 mm	0
≥16 mm	1
Patellar tilt	
≤20°	0
>20°	1
Total points	7

## Data Availability

The data presented in this study are available within the manuscript.
